# Pollution of the Niger Delta with total petroleum hydrocarbons, heavy metals and nutrients in relation to seasonal dynamics

**DOI:** 10.1038/s41598-023-40995-9

**Published:** 2023-08-28

**Authors:** Ihuoma N. Anyanwu, Sebastian Beggel, Francis D. Sikoki, Eric O. Okuku, John-Paul Unyimadu, Juergen Geist

**Affiliations:** 1 Department of Biology, Faculty of Biological Sciences, Alex Ekwueme Federal University Ndufu-Alike, P.M.B 1010, Abakaliki, Nigeria; 2 Aquatic Systems Biology Unit, TUM School of Life Sciences, Technical University of Munich, Muehlenweg 22, 85354 Freising, Germany; 3 Department of Animal and Environmental Biology, Faculty of Science, University of Port Harcourt, P.M.B.1023, Choba, Nigeria; 4 Kenya Marine and Fisheries Research Institute, P.O. Box 81651, Mombasa, Kenya; 5 Nigerian Institute for Oceanography and Marine Research, Victoria Island, P.O. Box 74304, Lagos, Nigeria

**Keywords:** Environmental sciences, Environmental impact

## Abstract

The African Niger Delta is among the world’s most important wetlands in which the ecological effects of intensive oil exploitation and global change are not well documented. We characterized the seasonal dynamics and pollution with total-petroleum-hydrocarbons (TPHs), heavy-metals (HMs) and nutrient-loads in relation to climate-driven variables. High TPH concentrations up to 889 mg/L and HMs up to 13.119 mg/L were found in water samples, with pronounced spatio-temporal variation throughout the year. HM pollution index and contamination factor indicate serious ecological and human health hazards, especially for Cd, Cu, Hg, and Ni. Significant differences in TPHs/HMs were observed between sites and seasons, with correlations between TPHs-HMs, and climate-variables and TPHs-HMs. Nutrient levels, turbidity, salinity, temperature, and SO_4_^2-^ were high and interlinked with the variability of TPHs/HMs being greatest during wet season. These findings suggest an urgent need for improved pollution control in the Niger Delta taking into account the observed spatio-temporal variation and the exacerbation of effects in light of climate change. Given the high levels of contamination, further assessments of exposure effects and bioaccumulation in biota should include future climate change scenarios and effects on humans who intensively depend on the system for drinking water, food supply and livelihood.

## Introduction

The Niger Delta region is one of the largest wetland systems worldwide and can be considered a biodiversity hotspot comprising the greatest diversity of aquatic species in Africa^1–3^. Several severe anthropogenic pressures exist in this region, increasing the vulnerability of the system by contamination, global environmental change, and a resulting rapid quality decline in the region’s fragile ecosystems^1–6^. Contamination has been of major concern in the region due to the associated (eco)toxicity, bioaccumulation, persistence and risks to biota including humans^3–6^. Fundamentally, estuarine regions of large river systems are known sinks of contaminated sediments and a source of contamination for the adjacent marine habitats. Although this situation can be observed in many estuarine regions worldwide^3,6–9^, the African Niger Delta is an example of how a broad range of unsupervised human activities directly impact contaminant levels. Due to their persistence, some of the contaminants of concern are the total petroleum hydrocarbons (TPHs) and heavy metals (HMs) resulting from the heavy oil exploitation within the Niger Delta system. TPHs and HMs are persistent, bioaccumulative, toxic and carcinogenic compounds widely distributed in the aquatic environment in areas of oil exploitation and mining, which can be seen here to a specific extent. Although they can originate from natural sources such as weathering and soil erosion, the sources in the Niger Delta are linked to anthropogenic activities as a major emission pathway by atmospheric deposition, crude oil spills, unregulated industrial emissions and other sources. This results in a dispersal of these substances into the water column, or deposition in sediments^10^ exceeding natural background levels. Despite this general understanding, there is no systematic assessment undertaken to relate the occurrence and dynamics of these pollutants in light of changing and extreme climatic conditions. Global warming is not only affecting surface temperature, but will also result in altered salinity gradients, increasing floods and altered hydrological regimes, which can significantly affect contaminant mobilization and distribution and therefore bioavailability^6,10^. In contrast, drought events and high evaporation rates can lead to increased concentration of pollutants^11^. Consequently, there are concerns that TPH and HM levels in the ecosystems and associated risks may be altered and exacerbated by hydro-climate variables^10^.

Most regional studies have focussed on spatial distributions, occurrence, source identification and risk assessment^12,13^, and less attention has been paid to a systematic understanding of TPH and HM distribution patterns within the Niger Delta system in relation to hydroclimatic drivers. This knowledge is essential to project how climate variation can potentially cause multiple and significant changes in the Niger Delta system. For instance, increasing temperature is known to influence contaminant mobilization, fate, transformation, and bioavailability^14^, thus directly affecting risk assessment. Also, contaminant partitioning in water can be affected by changes in climate-related factors. Further, climate-driven variables can affect contaminant deposition in the water column by altering their input from surface sources and run off, or by changing water chemistry^10^. Similarly, changes in water levels caused by extreme rainfall, drought, and temperature changes can intensify cycling of TPHs/HMs in ecosystems^10,15^. These effects have so far not been determined in the various habitats of the Niger Delta. Thus, knowledge of both climate-driven variations and anthropogenic activities on the Niger Delta ecosystems is important in assessing the current state and predicting future development of aquatic ecosystems in the region.

In addition to future predictions, an understanding of the direct and indirect impacts of climate-driven shifts on contaminant mobility is also essential for current risk assessment and the deduction of regulatory measures in the region. Therefore, this study investigated three representative coastal ecosystems, the Imo river (freshwater system), the Bonny estuary and the Lagos lagoon (brackish systems) within the Niger Delta over the course of one entire year. Water samples were analysed for metals including manganese (Mn), iron (Fe), aluminium (Al), copper (Cu), zinc (Zn), cobalt (Co), nickel (Ni), cadmium (Cd), lead (Pb), chromium (Cr), mercury (Hg), boron (B), barium (Ba), molybdenum (Mo), as well as total petroleum hydrocarbons (TPHs) from water samples, while in parallel, measuring the climate-associated variables (abiotic parameters) and nutrient levels phosphate (PO_4_^3−^), nitrate (NO_3_^−^) and ammonium (NH_4_^+^). This aimed at clarifying to which extent hydro-climate associated changes in the aquatic environment are interconnected to the mobilization of TPHs and HMs in the system. An additional objective was the identification of parameters related to climate sensitivity of TPHs/HMs, highlighting the possible effects on ecosystem health and implications for human life in the region. We hypothesized that (i) there are differences between the respective habitats investigated (site-specific profiles and contaminant occurrence), (ii) that the concentration and bioavailability of major contaminants is directly linked to seasonal variation and climate-hydrological variables, which (iii) requires inclusion of such information in risk assessment in light of global change affecting environmental variables such as temperature and salinity in the different ecosystems of the Niger Delta.

## Materials and methods

**Figure 1:**
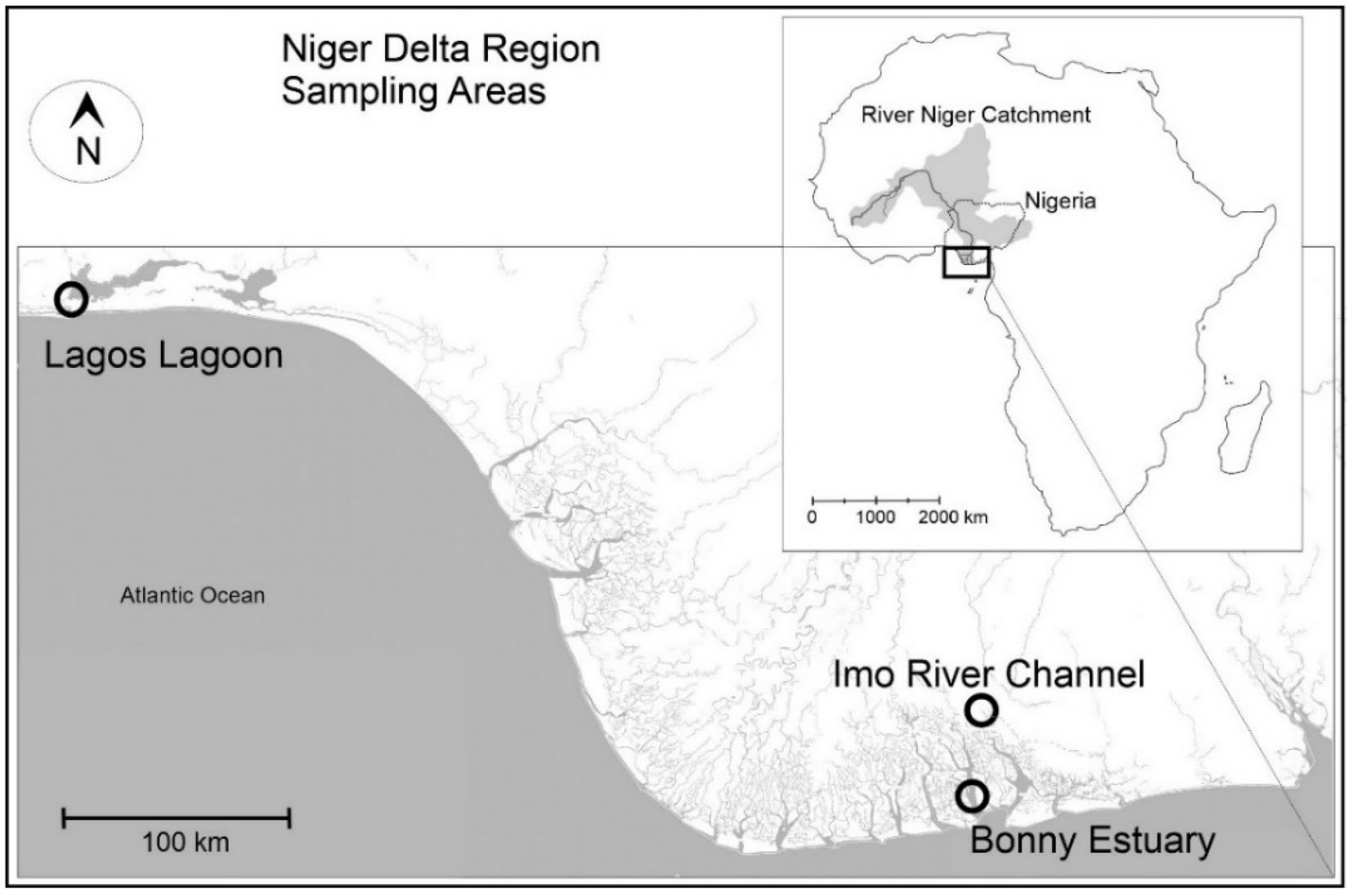
Map of the Niger Delta indicating sampling sites. The map was created using CorelDRAW 2020 (Version 22.0.0.412, Corel Corporation Inc.). Data was extracted from OpenStreetMap (https://www.openstreetmap.org), licensed on terms of the Open Database License, “ODbL” 1.0 (https://opendatacommons.org/licenses/odbl/), previously licensed CC-BY-SA 2.0.

Surface water samples were collected from the three major ecosystem types of the Niger Delta, the Bonny estuary, the Imo river and the Lagos lagoon (Fig. 1) over 1 year. Stations (3–4) representing upper, middle, lower reaches^16^ (> 5 km apart) were sampled in each site in the estuary, river, and lagoon. Monthly water samples were collected from the mapped stations over an entire annual cycle from January to December, 2021. TPHs and HMs (Mn, Al, Co, B, Ba, Zn, Cr, Cu, Ni, Pb, Cd, Hg) were measured in mg/L. In brief, TPHs: 5 mL samples were extracted with 50 mL Toluene in a separating funnel, and the aqueous layer was measured with UV–Vis spectrophotometer. HMs: 100 mL samples were evaporated, digested with 10 mL HNO_3_, and then 5 mL perchloric acid, and analysed with Atomic Absorption Spectrophotometer (Supplementary Data). The degree of anthropogenic metal contamination levels of the three habitat types was further determined by contamination factor (CF), and the cumulative factor was calculated as the mean ratio of measured sample concentrations and the reference (using the national fisheries and recreation quality standard and USEPA regulations) (see detailed description in the Supplementary Data). Pollution load index (PLI) for HMs in water was computed based on engine values > 1, and > 3 for Nemerow pollution index (NPI). Climate-related parameters temperature (°C), salinity (ppt), conductivity (mS/cm), DO (mg/L), TDS (mg/L), pH, were measured in-situ using a Horiba U-52 Multi-parameter meter, while, nutrients PO_4_^3−^ (mg/L), NO_3_^−^ (mg/L), NH_4_^+^ (mg/L), and SO_4_^2−^ (mg/L), turbidity (NTU)) were determined using established standard methods in APHA^17^ and Anyanwu et al.^16^ to understand the extent of hydro-climate associated changes in the systems.

### Data analysis

Data analysis was performed using Primer 7 software (Version 7.0.21, PRIMER-e, Quest Research Limited) for multivariate analysis and OriginPro (Version 2021, Originlabs) for visualization and significance testing of correlation coefficients. Analysis and characterization of patterns in multivariate data were carried out using Principal Component Analysis (PCA) based upon Euclidean distance, similarity profile (SIMPROF) plot, hierarchical clustering, permutation analysis (PERMANOVA) and distance based linear modelling (DistLM) with Primer 7. PCA estimated variations in TPHs/HMs mobilization and climate variables among sites, stations and seasons. SIMPROF plotted the pattern associated with TPHs/HMs within the 95% confidence limit. The multivariate analyses were used to identify the relevant abiotic/environmental parameters from the dataset that were associated with TPHs/HMs mobilization. PERMANOVA and DistLM were used to test for statistical significant differences in the dataset. In addition, Pearson’s correlation was used to explore linear relationships between contaminants and the environmental abiotic parameters. The degree of association was measured by a correlation coefficient (a measure of linearity or linear association). Data was inspected visually using Draftsman plots implemented in the Primer 7 software prior to analysis, and only linear relationships were considered valid for the discussion of results. Person correlations were calculated using OriginPro (Version 2021, OriginLab Corporation). Significance was accepted at p < 0.05.

## Results

### Contamination levels of TPHs and HMs in the Niger Delta coastal systems

High contamination levels of TPHs ranging from 17.38 to 889.10 mg/L (95.6–889.10 mg/L in estuary, 17.38–330.26 mg/L in river, 26.52–505.45 mg/L in lagoon), and of HMs ranging from 0 to 13.119 mg/L (0–3.65 mg/L in estuary, 0–4.722 mg/L in river, 0–13.119 mg/L in lagoon) with mean values higher than the national and USEPA regulatory standards were measured in the different habitats (Table 1). The estuary and upper reach of the lagoon (Ijora station) recorded elevated concentrations of TPHs (> 880 mg/L and > 500 mg/L) respectively. Oil exploitation and related port activities (including petroleum loading and off-loading), sewage and industrial discharge could be ascribed.

**Table 1:** Summary of TPH-HMs and environmental parameters in relation to existing ambient water quality criteria.

Chemical ambient water quality criteria for surface waters	Parameters														
	TPHs (mg/L)	Zn (mg/L)	Al (mg/L)	Cd (mg/L)	Cr (mg/L)	Cu (mg/L)	Fe (mg/L)	Pb (mg/L)	Hg (mg/L)	Ni (mg/L)	pH	PO_4_^3−^ (mg/L)	NO_3_^−^ (mg/L)	NH_4_^+^ (mg/L)	SO_4_^2−^ (mg/L)
Effluent discharge. Irrigation and reuse standards*	na	0.2	0.2	0.01	0.5	0.01	0.5	0.1	0.0005	0.1	6.5–8.5	3.5	40	2	500
Fisheries and recreation quality criteria standards*	na	0.01	0.2	0.005	0.5	0.001	0.05	0.01	0.001	0.001	6.5–8.5	3.5	9.1	0.05	100
USEPA freshwater CCC**	na	0.12		0.00072	0.011	na	1	0.0025	0.00077	0.052	6.5–9	na	na	na	na
USEPA saltwater CCC**	na	0.081		0.0079	0.05	0.0031	na	0.0081	0.00094	0.082	6.5–8.5	na	na	na	na
Overall mean Estuary stations	311.08	**0.020**	0.007	**0.017**	0.011	**0.094**	**0.102**	0.008	**0.002**	**0.113**	7.63	1.86	3.587	**5.55**	3.12
SD	147.22	0.023	0.009	0.022	0.014	0.138	0.147	0.020	0.003	0.163	0.47	1.70	4.531	7.33	3.31
Maximum	889.1	0.1	0.033	0.083	0.05	0.564	0.692	0.101	0.013	0.596	8.56	6.09	23.8	30.8	13.75
Overall mean River stations	105.789	**0.030**	0.021	**0.032**	0.018	**0.212**	**0.210**	0.003	**0.009**	**0.190**	8.78	1.83	2.954	**4.49**	4.26
SD	63.28	0.038	0.052	0.037	0.018	0.247	0.239	0.005	0.012	0.236	0.659	1.90	2.854	5.03	3.54
Maximum	330.26	0.184	0.31	0.141	0.063	0.873	0.965	0.02	0.045	0.896	10.22	6.11	14.16	29.4	12.29
Overall mean Lagoon stations	282.83	**0.029**	0.028	**0.039**	0.024	**0.247**	**0.206**	**0.012**	**0.009**	**0.226**	8.17	1.99	4.469	**8.09**	4.79
SD	128.82	0.041	0.062	0.043	0.024	0.267	0.241	0.039	0.017	0.307	0.91	2.33	5.449	10.62	5.56
Maximum	505.45	0.21	0.342	0.167	0.098	0.992	0.74	0.23	0.085	1.276	11.06	8.8	23.8	39.16	25.63

No regulatory values available for the parameters Mn, B, Ba, Co and Mo.

*https://standards.lawnigeria.com/2020/08/18/national-environmental-surface-and-groundwater-quality-control-regulations-2010/ (accessed Nov. 2022)^40^.

**https://www.epa.gov/wqc/national-recommended-water-quality-criteria-aquatic-life-criteria-table#table (accessed Dec. 2022)^41^.

Values presented as annual mean from different sites in the estuary, river and lagoon. Values highlighted in bold are exceeding at least one of the regulatory thresholds (*SD* standard deviation, *CCC* criterion continuous concentration, *na* no data available).

**Figure 2:**
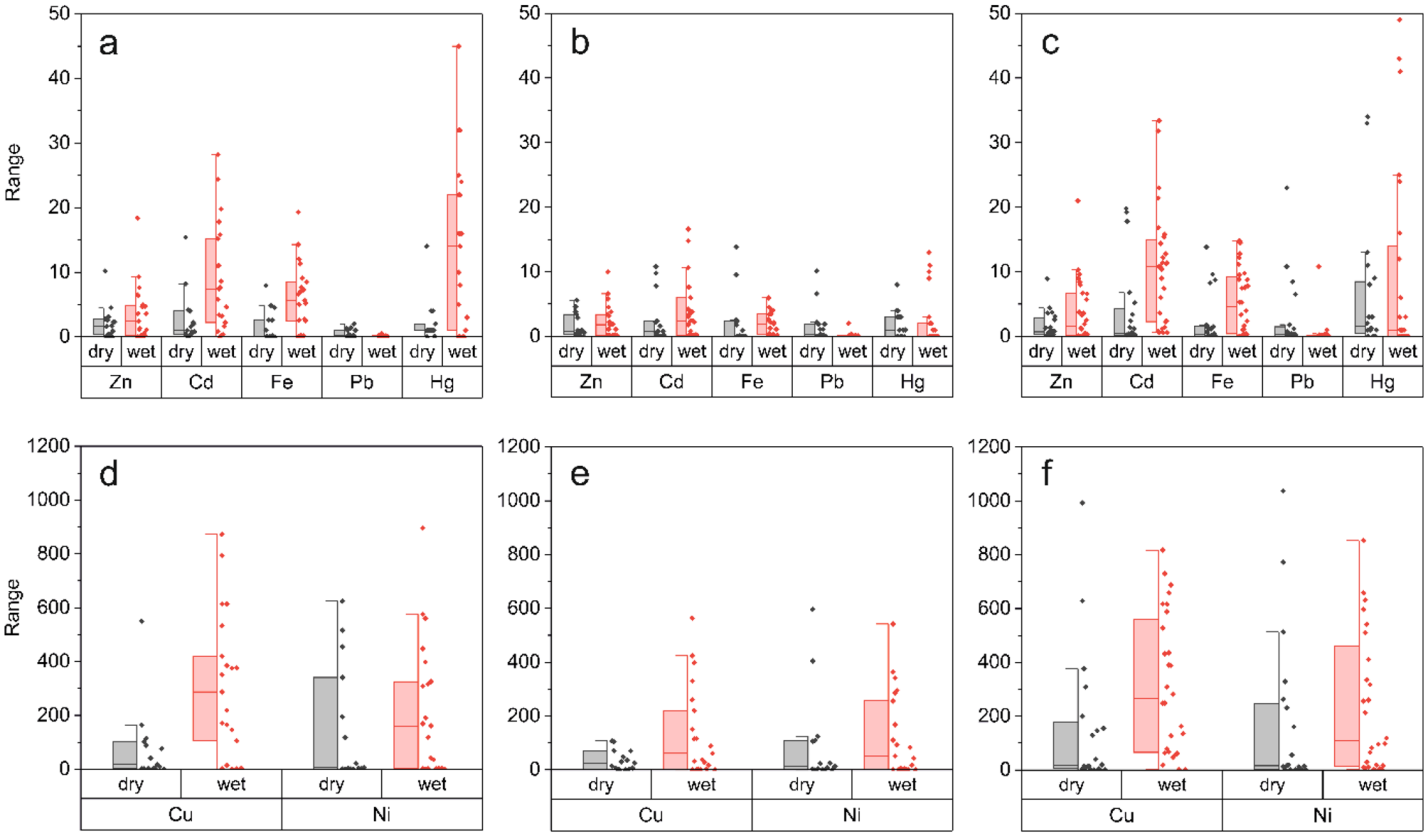
Combined box-scatterplot of contamination factors (CF) of TPHs and HMs in the Niger Delta coastal systems. CF calculation is based on the Fisheries and Recreation Quality Criteria Standards given by Nigerian law. CFs > 6 indicates a very high contamination. The box shows 25% to 75% range of values including seasonal median (–). Whiskers indicate the range of values within 1.5 inter-quartile ranges. Plots = Imo river (**a,d**), Bonny estuary (**b,e**), Lagos lagoon (**c,f**).

High levels of HM contamination were detected in the Niger Delta waters, particularly within the river and lagoon systems. The calculated CF values were 0.02–1.61 for Cr, 0.04–0.14 for Al, 0.17–3.04 for Zn, 0.10–4.19 for Fe, 0.25–3.16 for Pb, 2.18–226.21 for Ni, 2.36–11.65 for Hg, 3.48–44.48 for Cd, and 79.64–246.88 for Cu. This implies low to moderate levels of contamination for Zn, Al, and Cr, but considerable contamination for Pb, Fe and very high contamination for Ni, Cd, Cu, and Hg (Fig. 2, Table 1, Table S1).

The NPI and PLI were determined to assess the integrated pollution level at different sampling sites, as contaminants can significantly vary in various water samples. The calculated NPI values were 0.03–0.26 (Al), 0.04–1.14 (Cr), 0.14–2.16 (Zn), 0.18–2.23 (Pb), 0.49–3.04 (Fe), 1.67–8.24 (Hg), 2.46–31.45 (Cd), 1.60–159.96 (Ni), and 56.32–174.57 (Cu). PLI levels were 49.81–137.41 (estuary), 545.59–1505.17 (river) and 79.47–5593.25 (lagoon). Based on these values, greater toxicity and high ecological risk was observed in all the sampled ecosystems (Fig. 2, Table 1, Table S1).

**Figure 3:**
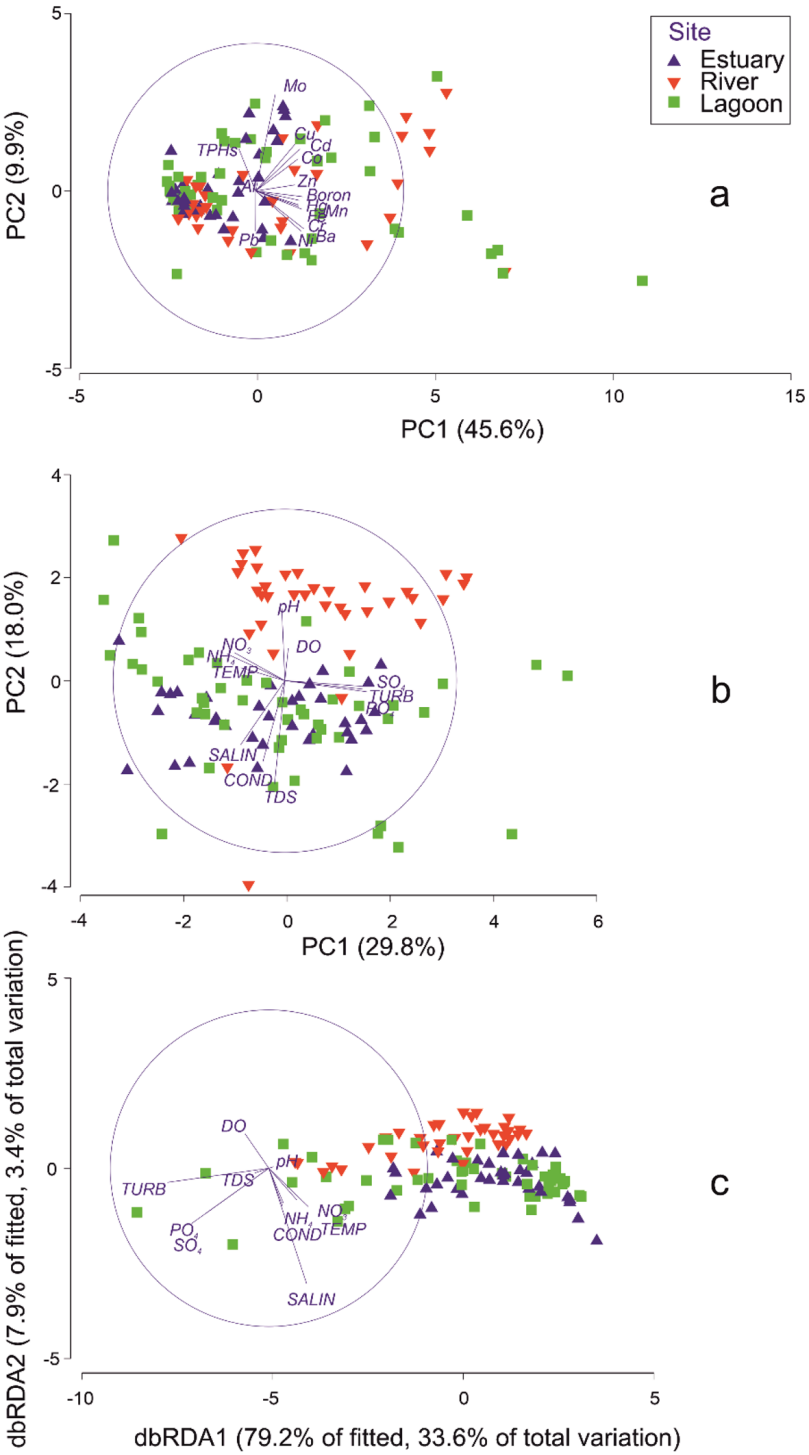
Principal component analysis of TPHs/HMs (**a**), climate parameters (**b**) and the distance based redundancy analysis biplots (dbRDA) (**c**) showing levels of variation, and the climate/environmental parameters responsible for TPHs and HMs mobilization in the Niger Delta coastal systems.

PCA analysis showed contaminant variation across sites, stations and seasons (Fig. 3). Ba, B, Cr, Ni, Fe, Cd, Hg (45.6%, PC 1) and Mo, Cu, TPHs, Pb, Co (9.9%, PC 2) mostly accounted for contaminant variation in the system (Fig. 3a). The dendrogram cluster hierarchy indicated that the contaminants have a common link and high degree of uniformity (R^2^ = 0.965; Fig. S1a). SIMPROF plot expressed interconnections and highly significant correlation (Pi = 0.813, p <0.01), signifying genuine relationship between the contaminants (Fig. S1b). This may suggest that the contaminants are associated with each other and/or emanate from similar sources.

**Figure 4:**
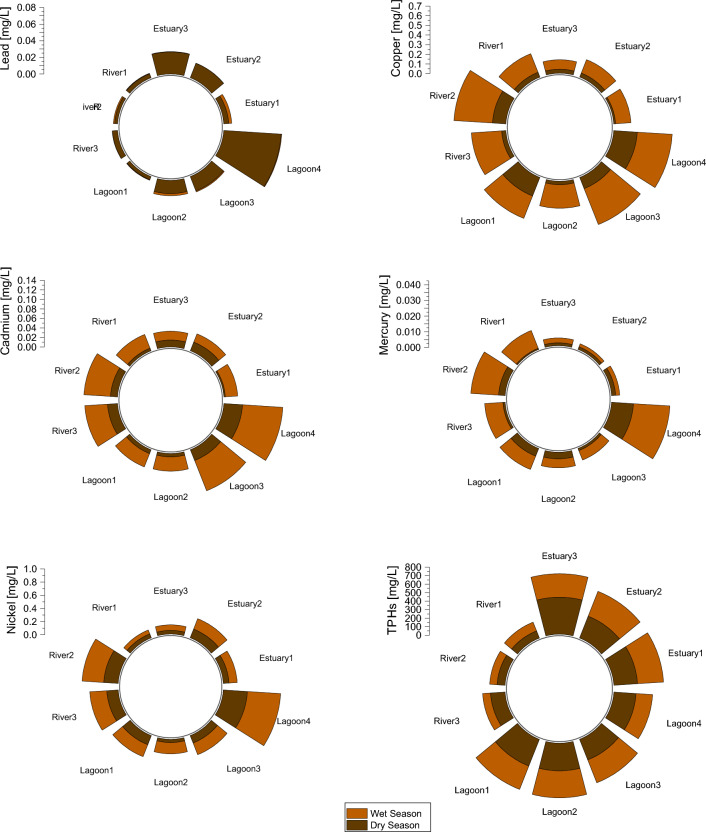
Distribution of TPHs and toxic HMs in the Niger Delta coastal systems.

Signature peaks and concentration variance occurred across sites and seasons (see also Fig. S2). Elevated concentrations of TPHs (70.1–889.1 mg/L), and Pb, Ba, Mn, Al (0.0–9.292 mg/L) were detected during dry season, while high levels of B, Cr, Fe, Hg, Ni, Zn, Cd, Co, Mo, Ba, and Cu were measured in the wet and early dry season months (0.0–13.119 mg/L) (Fig. 4, Fig. S3). However, contaminant concentrations for most HMs were higher during the dry season than wet season. High degrees of toxic metals (including Hg) were measured throughout the year, particularly in the river and lagoon systems (Fig. 4, Fig. S2b–e).

Further correlation test showed strong relationships specifically among the metals across sites (Table 3a–c) (p < 0.001, p < 0.01, p < 0.05), but there was no clear linear relationship between TPHs and metals for the different sites. Analogously, HM contamination levels were interlinked, except for Al and Pb in the estuary and lagoon system. On the other hand, they exhibited negative association with TPHs in the estuary.

### Influence of climate parameters on TPHs and HMs mobilization in the Niger Delta coastal systems

Throughout the annual hydrological cycle, physicochemical variables varied widely: water temperature ranged between 26.4 and 31.4 °C, DO from 1.8 to 6.6 mg/L, and conductivity between 16.74 and 79.12 mS/cm. pH was slightly acidic to alkaline (6.28–11.06), TDS was 11.03–81.00 mg/L, and turbidity ranged between 0.01 and 53.47 NTU. As expected, a salinity gradient from a low saline system (0.01 ppt) in the river to full saline environment (> 35 ppt) in brackish habitats was identified. Nutrients (PO_4_^3−^, NO_3_^−^, NH_4_^+^) were 0.01–8.25 mg/L, 0.08–23.80 mg/L and 0.32–35 mg/L, respectively, while sulphate (SO_4_^2−^) varied between 0.01 and 25.63 mg/L in the coastal system. Climate variables indicated that nutrients, SO_4_^2−^, turbidity (PC 1), and TDS, pH, salinity, conductivity (PC 2) mainly accounted for variation in the data (Fig. 3b). Linking contaminant patterns to climate parameters exhibited similarities in factors affecting TPHs/HMs distribution in various sites, seasons, stations, and the interaction term sites × seasons (p < 0.001, Table 2). PO_4_^3−^ and SO_4_^2−^ were found to be the most sensitive parameters related to TPHs/HMs mobilization in the region, while turbidity showed strong impact during the wet season (as a result of strong rain). PERMANOVA indicated that PO_4_^3−^, turbidity, conductivity, salinity, DO, temperature significantly affected TPHs and HMs mobility in all the study sites, seasons and the interaction term (sites × seasons) (p < 0.01). The regression analysis (DistLM) also confirmed that the climate-driven variables significantly influenced TPHs and HMs mobilization in the systems (p < 0.01) with the exception of conductivity, TDS and pH as displayed by the distance based redundancy analysis biplots (dbRDA) (Fig. 3c). However, temperature, salinity, PO_4_^3−^, SO_4_^2−^ and turbidity are the most sensitive parameters affecting contaminant mobilization in the African Niger Delta system.

**Table 2:** Statistical summary of environmental parameters influencing TPH-HMs mobilization in the Niger Delta coastal systems.

Variable measured	**Parameter**	R^2^	p-value
Site	PO_4_^3−^, SO_4_^2−^	0.446	0.001
Season	Turbidity, SO_4_^2−^	0.505	0.001
Stations	SO_4_^2−^	0.392	0.001
Site × season	SO_4_^2−^	0.461	0.001

Variables represent sites and different stations in the estuary, river, and lagoon.

Significant relationships among contaminants and between climate parameters and contaminants were evident in the river and lagoon systems (Table 3a, c). Correlation analysis showed a general strong relationship between climate parameters and contaminants across sites (Table 3a–c) (p < 0.001, p < 0.01, p < 0.05). An interrelation between turbidity, conductivity, pH, salinity, DO, PO_4_^3−^, and temperature on the one hand, and TPHs/HMs mobilization across sites and seasons on the other hand was identified. Temperature and conductivity correlated negatively with HMs at all sites. In the lagoon system, turbidity, PO_4_^3−^, SO_4_^2−^ and the HMs was inversely related to TPHs, whereas salinity and pH showed positive relationship with TPHs. Interestingly, temperature displayed both direct and indirect effects towards TPHs/HMs mobilization. Temperature had direct negative relationships with Zn, B, Cr, Fe, Hg (in river); Zn, B, Ba, Co, Ni (in estuary); Zn, B (in lagoon); and indirect link with climate parameters (including salinity, TDS, turbidity, DO, conductivity) responsible for TPHs/HMs mobilization in the ecosystems, particularly salinity (Table 3a–c). Temperature also showed strong link with nutrients (PO_4_^3−^, NH_4_^+^) in the estuarine system. In addition, climate variables such as salinity, TDS, pH, DO, turbidity and nutrients (NH_4_^+^, PO_4_^3−^) as well as SO_4_^2−^ interrelated with each other and were linked with TPH and HM mobilization (mostly within the freshwater part of the system).

**Table 3:** Pearson correlation coefficients between TPHs/HMs, and TPHs/HMs and environmental parameters in the Niger Delta coastal systems (lower left diagonal) and associated p-values (upper right diagonal).

	TPHs	Mn	Zn	Al	B	Cd	Ba	Cr	Co	Cu	Fe	Pb	Hg	Mo	Ni	Temp	DO	Cond	TDS	pH	Turb	Sal	PO_4_^3−^	NO_3_^−^	NH_4_^+^	SO_4_^2−^
a—River																										
TPHs	–	0.03	0.64	0.52	0.56	0.07	0.25	0.28	0.31	0.48	0.75	0.34	0.32	0.69	0.52	0.49	0.74	0.72	0.83	0.57	0.37	0.52	0.27	0.12	0.02	0.09
Mn	0.37	–	0.00	0.90	0.00	0.00	0.00	0.03	0.00	0.01	0.00	0.08	0.00	0.02	8.02	0.04	0.02	0.65	0.78	0.07	0.91	0.19	0.01	0.51	0.53	0.07
Zn	0.08	**0.59**	–	0.88	6.57	0.08	0.00	0.00	0.01	0.00	0.32	0.17	0.00	0.07	0.01	0.01	0.09	0.53	0.55	0.09	0.01	0.06	0.01	0.58	0.56	0.00
Al	− 0.11	− 0.02	− 0.03		0.60	0.92	0.67	0.61	0.84	0.55	0.51	0.03	0.74	0.66	0.62	0.08	0.45	0.44	0.46	0.30	0.63	0.83	0.58	0.66	0.88	0.49
B	0.10	**0.60**	**0.62**	− 0.09	–	0.00	5.03	0.03	0.00	8.10	0.00	0.05	0.18	0.00	0.00	0.01	0.02	0.81	0.63	0.22	0.00	0.18	0.00	0.08	0.10	0.00
Cd	0.30	**0.55**	0.29	0.02	**0.53**	–	0.00	0.00	0.01	2.49	0.02	0.21	0.03	0.10	0.04	0.20	0.22	0.28	0.36	0.68	0.01	0.12	0.00	0.48	0.21	0.01
Ba	0.20	**0.82**	**0.59**	− 0.07	**0.62**	**0.51**	–	0.00	0.00	0.00	1.39	0.16	0.00	0.08	0.00	0.02	0.00	0.60	0.69	0.00	0.00	0.07	0.00	0.16	0.10	0.00
Cr	0.19	**0.69**	**0.85**	− 0.09	**0.74**	**0.54**	**0.77**	–	0.00	0.00	0.01	0.09	0.01	0.01	0.00	0.00	0.03	0.20	0.21	0.02	0.00	0.03	5.32	0.54	0.29	0.26
Co	0.18	**0.68**	**0.43**	− 0.04	**0.60**	**0.45**	**0.50**	**0.54**	–	6.91	0.00	0.03	0.01	0.01	0.04	0.08	0.01	0.63	0.86	0.50	0.02	0.66	0.01	0.49	0.24	0.02
Cu	0.12	**0.76**	**0.47**	0.1	**0.61**	**0.64**	**0.53**	**0.54**	**0.61**	–	0.00	0.17	2.70	6.44	0.04	0.33	0.31	0.21	0.32	0.88	0.02	0.42	0.00	0.08	0.08	0.02
Fe	0.06	**0.60**	**0.69**	− 0.11	**0.84**	**0.40**	**0.66**	**0.77**	**0.58**	**0.56**	–	0.01	9.69	0.01	0.00	0.00	0.00	0.63	0.77	0.16	0.00	0.12	0.00	0.28	0.30	0.00
Pb	− 0.16	− 0.29	− 0.23	0.36	− 0.32	− 0.21	− 0.24	− 0.29	− 0.37	− 0.24	− **0.41**	–	0.05	0.18	0.18	0.26	0.01	0.38	0.38	0.08	0.21	0.44	0.10	0.89	0.96	0.17
Hg	0.17	**0.55**	**0.58**	− 0.06	**0.70**	**0.74**	**0.53**	**0.75**	**0.42**	**0.64**	**0.60**	− 0.33	–	0.15	0.12	0.01	0.04	0.20	0.21	0.35	0.05	0.01	0.00	0.37	0.33	0.01
Mo	0.07	0.38	0.31	0.08	**0.51**	0.72	0.30	**0.41**	**0.42**	**0.62**	**0.45**	− 0.23	**0.71**	–	0.63	0.90	0.17	0.43	0.57	0.35	0.86	0.01	0.23	0.71	0.64	0.37
Ni	0.11	**0.61**	**0.42**	− 0.09	**0.49**	0.35	0.82	0.56	0.34	0.34	**0.55**	− 0.23	0.27	0.08	–	0.02	0.00	0.65	0.74	0.00	0.00	0.68	0.89	0.11	0.07	0.00
Temp	− 0.12	− 0.35	− **0.43**	0.30	− **0.44**	− 0.22	− 0.38	− **0.48**	− 0.29	− 0.17	− **0.52**	0.2	− **0.43**	− 0.02	− 0.37	–	0.00	0.65	0.65	0.03	0.04	0.43	0.04	0.19	0.20	0.02
DO	− 0.06	− **0.40**	− 0.29	0.13	− 0.39	− 0.21	− **0.48**	− 0.37	− **0.42**	− 0.17	− **0.59**	**0.45**	− 0.34	− 0.24	− **0.48**	**0.48**	–	0.90	0.95	0.07	583.0	0.38	0.01	0.09	0.10	0.00
Cond	− 0.06	− 0.08	− 0.11	− 0.13	0.04	− 0.19	− 0.09	− 0.22	− 0.08	− 0.21	− 0.08	− 0.15	− 0.22	− 0.13	− 0.08	0.08	0.02	–	0.00	0.77	0.85	0.65	0.39	0.74	0.86	0.61
TDS	− 0.04	− 0.05	− 0.10	− 0.13	0.08	− 0.16	− 0.07	− 0.21	− 0.03	− 0.17	− 0.05	− 0.15	− 0.22	− 0.10	− 0.06	0.08	0.01	**0.99**	–	0.69	0.91	0.65	0.47	0.69	0.81	0.66
pH	0.10	0.30	0.29	− 0.18	0.21	0.07	**0.47**	0.38	0.12	0.03	0.24	− 0.30	0.16	− 0.16	**0.5**	− 0.36	− 0.3	− 0.05	− 0.07	–	0.00	0.44	0.00	0.66	0.46	0.00
Turb	0.16	**0.67**	**0.42**	− 0.08	**0.54**	**0.40**	**0.81**	**0.59**	0.39	0.39	**0.49**	− 0.22	0.33	0.03	**0.86**	− 0.35	− **0.45**	− 0.03	− 0.02	**0.51**	–	0.87	0.00	0.17	0.05	0.00
Sal	0.11	0.23	0.31	− 0.04	0.23	0.27	0.31	0.37	0.08	0.14	0.26	− 0.13	**0.41**	**0.42**	− 0.07	− 0.14	− 0.15	0.08	0.08	0.13	− 0.03	–	0.04	0.97	0.83	0.10
PO_4_^3^ −	0.19	**0.75**	**0.45**	− 0.1	**0.48**	**0.47**	**0.84**	**0.62**	**0.43**	**0.54**	**0.50**	− 0.28	**0.46**	0.21	**0.67**	− 0.35	− **0.44**	− 0.15	− 0.13	**0.55**	**0.81**	0.35	–	0.22	0.09	0.00
NO_3_ −	0.26	− 0.11	− 0.1	0.08	− 0.29	− 0.12	− 0.24	− 0.11	− 0.12	− 0.30	− 0.19	0.03	− 0.15	− 0.07	− 0.27	0.22	0.28	− 0.06	− 0.07	− 0.08	− 0.23	− 0.01	− 0.21	–	0.00	0.37
NH_4_^+^	0.38	− 0.11	− 0.10	0.03	− 0.28	− 0.21	− 0.28	− 0.18	− 0.20	− 0.30	− 0.18	0.01	− 0.17	− 0.08	− 0.31	0.22	0.28	− 0.03	− 0.04	− 0.13	− 0.32	− 0.04	− 0.28	**0.92**	–	0.19
SO_4_^2^	0.29	**0.72**	**0.53**	− 0.12	**0.49**	**0.46**	**0.90**	**0.69**	0.39	**0.40**	**0.52**	− 0.23	**0.44**	0.16	**0.79**	− 0.39	− **0.47**	− 0.09	− 0.08	**0.58**	**0.88**	0.28	**0.92**	− 0.16	− 0.22	–
b—Estuary																										
TPHs	–	0.14	0.16	0.24	0.05	0.36	0.14	0.11	0.79	0.58	0.09	0.56	0.36	0.37	0.25	0.90	0.89	0.22	0.21	0.59	0.91	0.14	0.29	0.68	0.78	0.27
Mn	− 0.25	–	0.66	0.99	0.90	0.10	0.40	0.71	0.37	0.38	0.01	0.16	0.78	0.85	0.71	0.89	0.77	0.02	0.02	0.14	0.21	0.67	0.54	0.06	0.04	0.88
Zn	0.24	− 0.08	–	0.20	0.00	0.03	0.00	0.00	0.01	0.70	0.03	0.14	0.00	0.09	0.07	0.01	0.51	0.35	0.32	0.05	0.05	0.04	0.00	0.11	0.05	0.00
Al	0.2	0.00	0.22	–	0.33	0.02	0.56	0.08	0.21	0.41	0.27	0.73	0.04	0.78	0.24	0.28	0.78	0.20	0.14	0.26	0.69	0.22	0.49	0.89	0.85	0.85
B	0.33	− 0.02	**0.51**	0.17	–	0.38	0.00	0.02	0.44	0.51	0.12	0.23	0.29	0.97	0.00	0.00	0.15	0.00	0.00	0.26	0.00	0.30	0.00	0.26	0.11	0.00
Cd	0.16	0.28	0.36	0.38	0.15	–	0.68	0.05	0.00	0.00	0.15	0.94	0.11	0.04	0.00	0.08	0.25	0.40	0.47	0.45	0.96	0.58	0.37	0.05	0.03	0.32
Ba	0.25	− 0.15	**0.5**	− 0.10	**0.60**	0.07	–	0.02	0.03	0.21	0.26	0.55	0.05	0.00	0.09	0.00	0.23	0.44	0.49	0.02	0.01	0.45	0.00	0.24	0.12	0.02
Cr	0.27	− 0.06	**0.58**	0.29	**0.40**	0.34	0.39	–	0.70	0.90	0.78	0.34	0.42	0.38	0.08	0.17	0.63	0.35	0.33	0.52	0.07	0.03	0.00	0.14	0.08	0.16
Co	0.05	0.16	**0.42**	− 0.21	0.13	**0.46**	0.36	0.07	–	0.00	0.00	0.10	0.27	0.00	0.15	0.00	0.59	0.58	0.52	0.00	0.38	0.32	0.02	0.04	0.02	0.10
Cu	0.09	0.15	0.07	− 0.14	− 0.11	**0.54**	0.21	− 0.02	**0.64**	–	0.91	0.28	0.32	0.00	0.22	0.13	0.07	0.51	0.52	0.11	0.64	0.96	0.12	0.39	0.39	0.63
Fe	− 0.28	**0.43**	0.36	− 0.19	0.27	0.24	0.19	0.05	**0.55**	0.02	–	0.00	0.40	0.97	0.26	0.03	0.31	0.00	0.00	0.25	0.06	0.14	0.49	0.16	0.11	0.16
Pb	− 0.10	0.24	0.25	− 0.06	0.21	− 0.01	0.1	− 0.17	0.28	− 0.19	**0.73**	–	0.98	0.21	0.80	0.68	0.64	0.16	0.19	0.24	0.29	0.24	0.53	0.69	0.77	0.47
Hg	0.16	0.05	**0.46**	0.34	0.18	0.27	0.32	0.14	0.19	0.17	0.15	− 0.01	–	0.01	0.53	0.88	0.27	0.36	0.31	0.89	0.87	0.36	0.55	0.23	0.49	0.48
Mo	0.16	− 0.03	0.28	− 0.05	0.01	0.35	**0.47**	0.15	**0.67**	**0.77**	− 0.01	− 0.21	**0.42**	–	0.41	0.11	0.61	0.08	0.09	0.01	0.93	0.67	0.03	0.68	0.44	0.45
Ni	0.20	0.06	0.30	0.20	**0.72**	**0.50**	0.29	0.30	0.25	0.21	0.19	− 0.04	0.11	0.14	–	0.00	0.79	0.00	0.01	0.96	0.00	0.30	0.00	0.17	0.05	0.00
Temp	0.02	− 0.02	− **0.41**	0.18	− **0.53**	− 0.30	− **0.47**	− 0.23	− **0.49**	− 0.26	− 0.36	− 0.07	− 0.03	− 0.27	− **0.54**	–	0.11	0.00	0.00	0.42	0.00	0.01	0.00	0.03	0.00	0.00
DO	− 0.02	0.05	− 0.11	0.05	− 0.25	0.20	− 0.20	− 0.08	0.09	0.30	− 0.17	− 0.08	− 0.19	0.09	0.05	0.27	–	0.27	0.28	0.81	0.34	0.18	0.40	0.73	1.00	0.38
Cond	0.21	− **0.40**	− 0.16	0.22	− **0.48**	− 0.14	− 0.13	− 0.16	− 0.1	0.11	− **0.50**	− 0.24	0.16	0.30	− **0.47**	**0.68**	0.19	–	0.00	0.73	0.00	0.01	0.01	0.36	0.14	0.00
TDS	0.22	− 0.39	− 0.17	0.25	− **0.46**	− 0.13	− 0.12	− 0.17	− 0.11	0.11	− **0.48**	− 0.22	0.17	0.29	− **0.45**	**0.70**	0.19	**0.99**	–	0.82	0.00	0.01	0.00	0.40	0.16	0.00
pH	− 0.09	− 0.25	− 0.33	0.19	− 0.19	− 0.13	− **0.40**	− 0.11	− **0.50**	− 0.27	− 0.20	− 0.20	− 0.03	− **0.45**	0.01	0.14	0.04	− 0.06	− 0.04	–	0.64	0.90	0.73	0.30	0.55	0.65
Turb	0.02	0.22	0.34	− 0.07	**0.58**	0.01	**0.43**	0.31	0.15	− 0.08	0.32	0.18	− 0.03	− 0.02	0.55	− **0.49**	− 0.16	− **0.53**	− **0.53**	− 0.08	–	0.28	0.01	0.59	0.60	0.00
Sal	0.25	− 0.07	− 0.35	0.21	− 0.18	− 0.10	− 0.13	− 0.36	− 0.17	0.01	− 0.25	0.2	− − 0.16	− − 0.07	− 0.18	**0.45**	0.23	**0.41**	**0.44**	0.02	− 0.19	–	0.00	0.32	0.08	0.12
PO_4_^3−^	0.18	− 0.11	**0.49**	− 0.12	**0.53**	0.16	**0.49**	**0.53**	0.39	0.27	0.12	− 0.11	0.10	0.36	**0.48**	− **0.76**	− 0.14	− **0.45**	− **0.47**	− 0.06	**0.43**	− **0.50**	–	0.14	0.02	0.00
NO_3_^−^	0.07	0.32	− 0.27	0.02	− 0.19	− 0.32	− 0.2	− 0.25	− 0.35	− 0.15	− 0.24	− 0.07	0.20	− 0.07	− 0.23	0.37	− 0.06	0.16	0.14	0.18	0.09	0.17	− 0.25	–	0.00	0.49
NH_4_^+^	0.05	0.34	− 0.33	0.03	− 0.27	− 0.36	− 0.27	− 0.29	− 0.39	− 0.15	− 0.27	− 0.05	0.12	− 0.13	− 0.32	**0.51**	0.00	0.25	0.24	0.10	0.09	0.30	− 0.38	**0.94**	–	0.16
SO_4_^2^	0.19	0.03	**0.48**	0.03	**0.79**	0.17	0.37	0.24	0.28	0.08	0.24	0.12	0.12	0.13	**0.77**	− **0.60**	− 0.15	− **0.52**	− **0.51**	− 0.08	**0.56**	− 0.26	**0.68**	− 0.12	− 0.24	–
c—Lagoon																										
TPHs	–	0.00	0.01	0.11	0.00	0.02	0.00	0.00	0.00	0.01	0.00	0.29	0.02	0.65	0.00	0.20	0.11	0.09	0.35	0.00	0.00	0.00	0.00	0.15	0.09	0.00
Mn	− **0.46**	–	0.01	0.32	0.00	0.00	0.00	0.00	0.00	0.00	0.00	0.93	0.00	0.82	0.00	0.76	0.06	0.06	0.73	0.55	0.00	0.00	0.00	0.03	0.09	0.00
Zn	− 0.35	0.35	–	0.22	0.00	0.00	0.00	0.00	0.00	0.00	0.00	0.92	0.00	0.12	0.00	0.00	0.88	0.32	0.01	0.04	0.00	0.00	0.00	0.10	0.09	0.02
Al	0.23	− 0.15	− 0.18	–	0.31	0.27	0.17	0.42	0.21	0.17	0.27	0.77	0.73	0.88	0.65	0.13	0.65	0.24	0.53	0.35	0.56	0.01	0.56	0.43	0.58	0.45
B	− **0.54**	**0.56**	**0.54**	− 0.15	–	0.00	0.00	0.00	0.00	0.00	0.00	0.68	0.00	0.20	0.00	0.00	0.36	0.14	0.01	0.03	0.00	0.00	0.00	0.07	0.02	0.00
Cd	− 0.34	**0.48**	**0.60**	− 0.16	**0.60**	–	0.00	0.00	0.00	0.00	0.00	0.60	0.00	0.00	0.00	0.08	0.81	0.00	0.76	0.20	0.00	0.00	0.00	0.01	0.01	0.00
Ba	− **0.60**	**0.62**	**0.52**	− 0.2	**0.70**	**0.61**	–	0.00	0.00	0.00	0.00	0.71	0.00	0.18	0.00	0.24	0.23	0.07	0.34	0.00	0.00	0.00	0.00	0.09	0.04	0.00
Cr	− **0.52**	**0.66**	**0.52**	− 0.12	**0.69**	**0.68**	**0.86**	–	0.00	0.01	0.00	0.81	0.00	0.63	0.00	0.33	0.23	0.03	0.70	0.04	0.00	0.00	0.00	0.04	0.01	0.00
Co	− **0.42**	**0.54**	**0.51**	− 0.19	**0.59**	**0.82**	**0.6**	**0.58**	–	0.00	0.00	0.81	0.00	0.03	0.00	0.22	0.74	0.01	0.67	0.02	0.00	0.00	0.00	0.02	0.01	0.00
Cu	− 0.38	**0.45**	**0.45**	− 0.2	**0.51**	**0.63**	**0.43**	0.36	**0.57**	–	0.00	0.65	0.02	0.14	0.01	0.01	0.01	0.05	0.46	0.06	0.01	0.00	0.03	0.01	0.01	0.04
Fe	− **0.65**	**0.55**	**0.43**	− 0.16	**0.67**	**0.46**	**0.53**	**0.51**	**0.59**	**0.46**	–	0.60	0.00	0.30	0.00	0.13	0.07	0.48	0.12	0.03	0.00	0.00	0.00	0.02	0.01	0.00
Pb	− 0.16	− 0.01	− 0.01	− 0.04	− 0.06	− 0.08	0.05	− 0.04	0.04	− 0.07	0.08	–	0.82	0.43	0.97	0.46	0.04	0.11	0.22	0.16	0.79	0.99	0.85	0.71	0.84	0.97
Hg	− 0.33	**0.48**	**0.49**	− 0.05	**0.63**	**0.55**	**0.77**	**0.62**	**0.58**	0.34	**0.48**	− 0.03	–	0.31	0.00	0.18	0.68	0.70	0.04	0.09	0.00	0.00	0.00	0.53	0.44	0.00
Mo	0.07	− 0.03	0.23	− 0.02	0.19	**0.43**	− 0.2	0.07	0.31	0.21	0.15	− 0.12	− 0.15	–	0.57	0.01	0.99	0.10	0.46	0.21	0.31	0.04	0.41	0.10	0.16	0.27
Ni	− **0.60**	**0.72**	**0.46**	− 0.07	**0.78**	**0.63**	**0.87**	**0.87**	**0.66**	0.39	**0.66**	0.01	**0.79**	− 0.08	–	0.56	0.28	0.11	0.38	0.02	0.00	0.00	0.00	0.07	0.03	0.00
Temp	0.19	− 0.05	− **0.47**	0.22	− **0.47**	− 0.25	− 0.17	− 0.14	− 0.18	− 0.36	− 0.22	0.11	− 0.20	− **0.40**	− 0.09	–	0.92	0.35	0.00	0.08	0.82	0.00	0.50	0.64	0.43	0.87
DO	− 0.23	0.28	0.02	− 0.07	0.14	0.04	0.18	0.18	0.05	0.37	0.27	− 0.30	− 0.06	0.00	0.16	0.02	–	0.61	0.45	0.49	0.16	0.71	0.10	0.45	0.22	0.07
Cond	0.25	− 0.27	− 0.15	0.17	− 0.22	− **0.41**	− 0.26	− 0.31	− 0.39	− 0.29	− 0.11	− 0.23	− 0.06	− 0.24	− 0.23	− 0.14	− 0.08	–	0.00	0.51	0.17	0.03	0.21	0.57	0.78	0.14
TDS	− 0.14	− 0.05	0.39	0.09	0.36	0.05	0.14	0.06	0.06	0.11	0.23	− 0.18	0.30	0.11	0.13	− **0.67**	− 0.11	**0.43**	–	0.21	0.56	0.00	0.61	0.24	0.41	0.89
pH	**0.42**	− 0.09	− 0.30	0.14	− 0.32	− 0.19	− **0.43**	− 0.30	− 0.33	− 0.28	− 0.31	− 0.21	− 0.25	0.18	− 0.34	0.25	− 0.10	0.10	− 0.19	–	0.02	0.01	0.00	0.16	0.00	0.02
Turb	− **0.65**	**0.70**	**0.42**	− 0.09	**0.72**	**0.58**	**0.89**	**0.89**	**0.59**	0.36	**0.66**	0.04	**0.72**	− 0.15	**0.95**	− 0.03	0.21	− 0.20	0.09	− 0.33	–	0.00	0.00	0.05	0.02	0.00
Sal	**0.58**	− **0.43**	− **0.66**	0.34	− **0.82**	− **0.63**	− **0.66**	− **0.66**	− **0.58**	− **0.56**	− **0.61**	0.00	− **0.57**	− 0.29	− **0.66**	**0.64**	− 0.06	0.30	− 0.37	0.35	− **0.59**	–	0.00	0.26	0.09	0.00
PO_4_^3−^	− **0.48**	**0.69**	**0.40**	− 0.09	**0.69**	**0.50**	**0.83**	**0.86**	**0.54**	0.30	**0.54**	− 0.03	**0.70**	− 0.12	**0.92**	− 0.1	0.24	− 0.18	0.08	− 0.38	**0.90**	− **0.58**	–	0.07	0.02	0.00
NO_3_^−^	0.21	− 0.31	− 0.24	0.12	− 0.27	− 0.38	− 0.25	− 0.29	− 0.34	− 0.39	− 0.33	0.06	− 0.09	− 0.24	− 0.27	0.07	− 0.11	− 0.08	− 0.17	0.20	− 0.28	0.17	− 0.27	–	0.00	0.14
NH_4_^+^	0.24	− 0.25	− 0.25	0.08	− 0.33	− 0.39	− 0.29	− 0.35	− 0.35	− 0.39	− 0.35	0.03	− 0.12	− 0.21	− 0.32	0.12	− 0.18	− 0.04	− 0.12	**0.41**	− 0.34	0.25	− 0.33	**0.89**		0.05
SO_4_^2^	− **0.52**	**0.72**	0.35	− 0.11	**0.64**	**0.53**	**0.86**	**0.87**	**0.59**	0.30	**0.46**	− 0.01	**0.64**	− 0.16	**0.90**	− 0.02	0.26	− 0.21	0.02	− 0.33	**0.91**	− **0.52**	**0.89**	− 0.22	− 0.29	–

Correlation values = 0.00–0.10 (negligible correlation), 0.10–0.39 (weak correlation), 0.40–0.69 (moderate correlation), 0.70–0.89 (strong correlation), 0.90–1.00 (very strong correlation).

p < 0.0001, p < 0.01, p < 0.05.

Significant Pearson's r values are indicated in bold.

## Discussion

This present study is to our knowledge the first one that systematically characterised contaminant load and mobilization in relation to climate-driven variables over a one-year time course covering several sites in three main ecosystem types in the Niger Delta. The findings suggest severe contamination problems relating to hydro-climate associated changes in all three investigated ecosystems of the region concerning HMs, TPHs, and nutrients.

Results revealed that TPH/HM concentrations exceeded regulatory limits (Fig. 2, Table S1), and Cd, Cu, Hg, Ni showed very high contamination levels in the aquatic habitats. The observed values are higher than those previously measured in the lagoon^18^ and Woji creek^12,13^. This is of specific concern since the substances comprise bioaccumulative, persistent and toxic characteristics. It is widely known that Cd, Cu, Hg, and Ni are highly toxic metals linked to environmental problems and human health^19,20^. They can affect metabolic processes of aquatic organisms, and accumulation in humans can increase the risks of endocrine disruption, adverse reproductive effects, cancers, diseases of the lungs, digestive tract, skin, and may also affect the hematopoietic/immune systems and induce neurologic and reproductive toxicity^19,20^. It is obvious that anthropogenic activities associated with crude oil spills, industrial waste and other processes (flooding, petroleum combustion, gas flaring, shipping activities, petroleum loading and off-loading) are causing high TPHs/HMs deposition in the Niger Delta system, and that climate-driven changes will further increase the intensity of these contaminant problems. This also illustrates that the Niger Delta is now a problematic hotspot of high Cd, Cu, Hg and Ni contamination. Thus, ensuring availability and sustainable management of water and sanitation for all, with targets concerning water quality, water-use efficiency, and water resources management (SDG-6)^21^ should be prioritized so as to achieve the United Nations Sustainable Development Goals by 2030.

The observed intra-/inter-contaminant correlations indicate that TPHs-HMs mobilization is interrelated, and this highlights the important roles of climate parameters in contaminants cycling in the system. Correlations between variables may suggest similar levels of TPHs-HM contaminants, similar release patterns / sources of pollution, and associated dependence during their mobilization and re-mobilization in the aquatic environments. Also, the high correlation coefficient between contaminants and climate parameters suggests similar behaviour under similar climatic/environmental conditions.

Climate parameters are known to affect the fate, enrichment, speciation, transformation, and bioavailability of contaminants in the environment. Here, temperature directly or indirectly shapes TPHs/HMs mobilization. It is widely accepted that increase in temperature in environmental systems will increase the fate and transformation rate of contaminants. Increased mobilization and distribution may lead to elevated levels of TPHs and HMs, and increased potential exposure risk for aquatic communities and humans^14^. Theoretically, flow conditions, pH, temperature shifts, as well as salinity gradients and turbidity are known drivers for the mobilization/distribution of contaminants, especially heavy metals^22,23^. These parameters are likely to be altered under the changing climatic conditions and alterations in temperature and rainfall pattern for example could result in shifts of mean discharge, in seasonality and hydrological extremes such as floods or droughts. Further, spatial differentiated warming of 0.3–0.7 °C, rainfall redistribution, drought, and excessive rainstorms are being predicted for the African continent^24^, with the most extreme temperature increases up to 6–8 °C for the projection period 2070–2099^25^ predicted for parts of the Niger system. As a direct result, an increase in temperature has been demonstrated to increase the uptake and toxicity of dissolved metals and other contaminants for aquatic organisms^26^.

Other important factors are the adsorption and desorption processes of metals and hydrophobic contaminants in relation to turbidity, and our study recorded increased turbidity resulting from flooding due to increased particle transport and run-off. In the present data, we found elevated contaminant concentrations during the wet season, which are clearly associated with the mobilization effects. This study also showed that TPH/HM contaminants were highly variable during wet season, indicating that rainfall patterns resulted in changes of their mobility and distribution. Mobilization/re-mobilization of contaminants has been mentioned to occur more often during the wet seasons. Rothwell et al.^27^ shown that the fate of heavy metals could be promoted by high rain through modulated base-flow and storm-flow metal concentrations. Conversely, in our data set, higher concentrations were recorded in the dry season which can probably be explained by concentration effects. However, the projected risk of major floods are extreme for the Niger catchment, which will significantly affect contaminant dynamics in the Delta region. In light of climate change, more extreme events of low flow (resulting in higher contaminant concentration) and extreme floods (resulting in greater mobilization) are likely. We thus propose that such scenarios are considered in risk assessment.

TPHs-HMs mobilization was also affected by salinity, which can be seen in the gradient from the freshwater to brackish water sampling sites. Studies have shown that solubility of contaminants depends on salinity of the ambient water^28,29^. Acosta et al.^30^ observed that salinity increased heavy metal mobilization, as an increase in the salinity is associated with an increase in the concentrations of major cations (Na, K, Ca, Mg) that compete with heavy metals for the sorption sites on particulate matter and sediments. Due to increasing seawater intrusion at rising sea levels, reduced freshwater flows (both subsurface and surface) and geomorphologic alterations of coastlines, salinity levels are expected to increase in river deltas globally^31,32^, with the resulting hypersalinization and so-called “reverse estuary” conditions already described for river estuaries in West Africa^33^. Consequently, this effect will likely also exacerbate the challenges associated with the already existing pollution in the Niger Delta system.

Additionally, our data showed that anthropogenic activities and climate-driven variables are increasing turbidity, nutrient and SO_4_^2−^ loading into the aquatic ecosystems and this is significantly interlinked with contaminant mobilization in the area. Sadly, the complex interaction between nutrients, salinity, temperature, turbidity, SO_4_^2−^ and other factors observed in this study will further accelerate TPHs-HMs mobilization/re-mobilization, increasing problematic impacts on ecosystem health and humans due to higher exposure levels. Furthermore, the ability of organisms to cope with the additional stress under the TPHs/HMs-changing climate regime will differ among species and result in a modified transfer of contaminants within the food web.

From a human health perspective, it needs to be mentioned that residents are consuming aquatic species and drinking water from these ecosystems and/or use surface water for crop irrigation. Taking into account the elevated contaminant concentrations in this study, a significant exposure of the human population can be assumed via the above mentioned uptake routes^34,35^. Recently, University of Port Harcourt Hospital (in Niger Delta region) reported over 4000 cancer cases, with 313 developing blood cancer or leukemia^36^. The causes are currently unknown but could possibly be related to the very high contamination levels of Cd, Cu, Hg, and Ni observed in this study. These metal contaminants are known carcinogens significantly associated with lung, liver, esophagus, gastric, gallbladder, colon, rectum, breast, prostate, kidney, lymphatic and/or hematopoietic cancers^37–39^. Additionally, exposure to Ni has been linked with acute myeloid and lymphoblastic leukemia^37^, suggesting the potential role of Ni in the development of acute leukemia in the region. Our study highlights the dangers associated with the changing climate and pollution in the Niger Delta. Whilst this study cannot solve the problems, the documentation and characterization of a problem is a first step towards its solution.

## Conclusions

Our study identified a significant pollution problem related to TPHs/HMs and nutrients in the African Niger Delta. It also characterized the impact of climate and hydrological variables on exposure risk for wildlife and humans. The results show that TPHs/HMs load and mobilization depends on climate parameters and other environmental conditions such as temperature and salinity, which will both likely increase in light of global change. The findings also suggest that the ongoing oil exploitation and associated effects, as well as the interrelation with climate change may further exacerbate the problems in the future. Hence, there is an urgent need for improved pollution control and climate change measures in the Niger Delta taking into account different types of pollutants and their interaction with climate-driven variables, to enable us grow ‘the ecosystems we need for the future we want’.

### Supplementary Information

Supplementary Information.

## Data Availability

The authors confirm that the data supporting the findings of this study are available within the article and its Supplementary Materials. All raw data are available on the PANGAEA Data Publisher database (https://www.pangaea.de/).
