# Supplementary material for: Pollution of the Niger Delta with total petroleum hydrocarbons, heavy metals and nutrients in relation to seasonal dynamics
Source: Sci Rep. 2023 Aug 28;13:14079. doi: 10.1038/s41598-023-40995-9 (PMC10462702; doi:10.1038/s41598-023-40995-9)
Supplement: Supplementary file 1 — Supplementary Information. [file 41598_2023_40995_MOESM1_ESM.docx]

**Supplementary data**

**Table S1.** Calculated Contamination factor (CF), Nemerow pollution index (PI) and pollution load index (PLI) of heavy metals in the Niger Delta coastal systems

| Index | Ecosystem | Zn | Al | Cd | Cr | Cu | Fe | Pb | Hg | Ni |  | PLI |  |
| --- | --- | --- | --- | --- | --- | --- | --- | --- | --- | --- | --- | --- | --- |
| CF (National) | Estuary | 2.05 | 0.04 | 3.48 | 0.02 | 93.64 | 2.03 | 0.79 | 2.36 | 113.48 |  | 137.41 |  |
|  | River | 3.04 | 0.11 | 6.41 | 0.04 | 211.92 | 4.19 | 0.25 | 8.97 | 190.25 |  | 1505.17 |  |
|  | Lagoon | 2.89 | 0.14 | 7.88 | 0.05 | 246.88 | 4.12 | 1.20 | 9.09 | 226.21 | < 1 (low contamination) | 5593.25 |  |
| CF (USEPA) | Estuary | 0.17 | 0.04 | 24.19 | 1.04 | 93.64 | 0.10 | 3.16 | 3.07 | 2.18 | 1 ≤ < 3 (moderate contamination) | 49.81 |  |
|  | River | 0.25 | 0.11 | 44.48 | 1.61 | 211.92 | 0.21 | 1.00 | 11.65 | 3.66 | 3 ≤ < 6 (considerable contamination) | 545.59 | PLI < 1 (unpolluted system) |
|  | Lagoon | 0.36 | 0.14 | 4.99 | 0.48 | 79.64 | 0.21 | 1.48 | 9.67 | 2.76 | ≥6 (very high contamination) | 79.47 | PLI > 1 (polluted system) |
| NPI (National) | Estuary | 1.45 | 0.03 | 2.46 | 0.04 | 66.22 | 1.52 | 0.56 | 1.67 | 80.24 |  |  |  |
|  | River | 2.16 | 0.23 | 4.53 | 0.05 | 149.85 | 3.04 | 0.18 | 6.34 | 134.53 | PI ≤ 0.7 (grade security) |  |  |
|  | Lagoon | 2.05 | 0.26 | 5.57 | 0.08 | 174.57 | 2.96 | 0.86 | 6.43 | 159.96 | 0.7 ≤ PI < 1 (minimal impact) |  |  |
| NPI (USEPA) | Estuary | 0.14 | 0.03 | 17.10 | 0.74 | 66.22 | 0.49 | 2.23 | 2.17 | 1.60 | 1 ≤ PI < 2 (small impact) |  |  |
|  | River | 0.22 | 0.23 | 31.45 | 1.14 | 149.85 | 0.70 | 0.71 | 8.24 | 2.66 | 2 ≤ PI < 3 (moderate impact) |  |  |
|  | Lagoon | 0.29 | 0.26 | 3.53 | 0.34 | 56.32 | 0.54 | 1.06 | 6.84 | 2.15 | PI > 3 (heavy Impact) |  |  |

CF, NPI, PLI (National) = Contamination factor (CF), Nemerow pollution index (NPI) and pollution load index (PLI) using the national-environmental-surface-and-groundwater-quality-control-regulations-2010/ (accessed Nov. 2022).

CF, NPI, PLI (USEPA) = Contamination factor (CF), Nemerow pollution index (NPI) and pollution load index (PLI) using the epa.gov national-recommended-water-quality-criteria-aquatic-life-criteria-table (accessed Dec. 2022).

**a**

**b**

**Figure S1.** Dendrogram cluster sets (**a**) and Similarity profile (SIMPROF) (**b**) of TPHs and HMs in the Niger Delta coastal systems

**Figure S2.** Signature peaks of TPHs and HMs distribution in the Niger Delta coastal systems


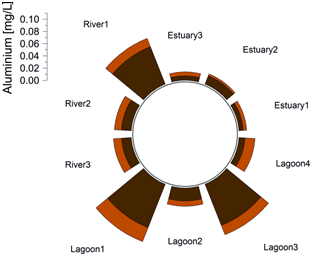

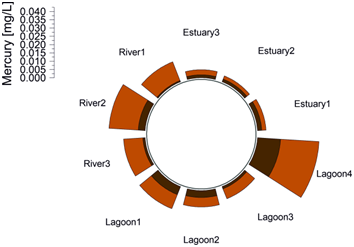

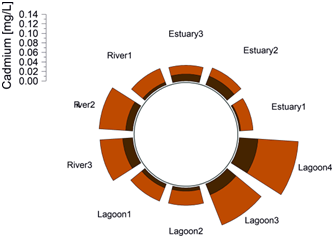

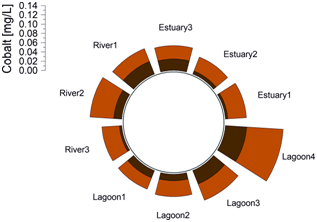

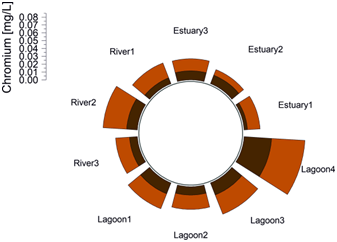

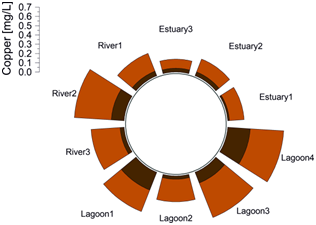

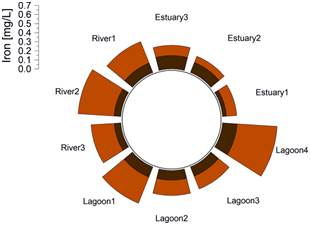

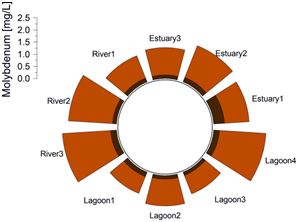

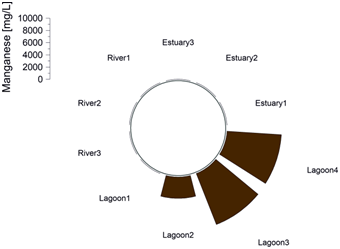

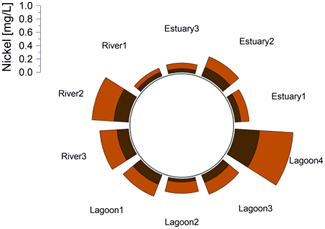

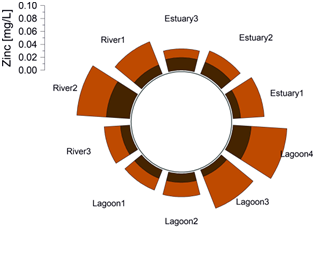

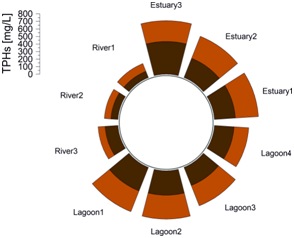

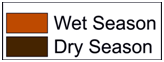

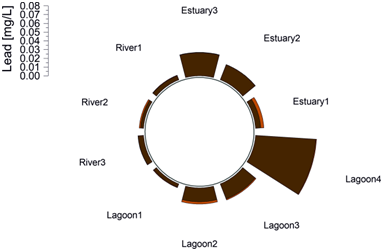

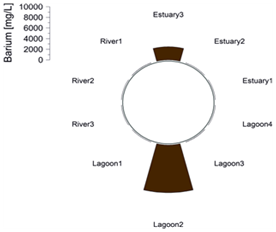


**Figure S3.** Spatio-temporal distribution of TPHs and HMs in the Niger Delta coastal systems

**Sample analysis**

***Total petroleum hydrocarbons (TPHs)***

TPHs extraction method was adapted from Lau et al. (2010). Water sample (5 mL) was transferred into 250 mL conical flask, 50 mL Na_2_SO_4_ solution was added, heated at 80^o^C for 1 hr over a water bath, and filtered with No 42 Whatman filter paper into a beaker. The residue was transferred into an extraction bottle containing 50 mL TPHs extracting solution, shaken 2 hrs using mechanical shaker, and filtered again into 50 mL volumetric flask. The filtrate was transferred into the extraction tube, 50 mL Toluene was added and shaken for 30 mins. This was then transferred into a separating funnel for separation. Aqueous layer was collected and measured at 350nm absorbance using UV/VIS spectrophotometer. Target compound concentration (mg/kg) was calculated from a calibration curve of standards (10 – 50 ppm) prepared in hexane measured at the same wavelength (350nm).

***Heavy metals (HMs)***

Sample (100 mL) was transferred into a beaker and placed on a hot plate to evaporate to dampness. Concentrated (70%) HNO_3_ (10 mL) was added, heated to near dryness, and 5 mL perchloric acid (60%) was added to complete the digestion. HMs in the sample digest was determined using Atomic Absorption Spectrophotometer (PG500 AAS instrument, BUCK Scientific, USA).

***Index calculation***

The metal contamination index $C_{f}^{i}$, pollution index (PI) and pollution load index (PLI) were calculated according to Formulas 1, 2, and 3. Modified after the description in Iordache et al. (2022). $C^{i}$ represents the mean measured metal concentration at the sampling site. $C_{n}^{i}$ should represent a pre-industrial or natural background concentration, which is not available for the Niger Delta. Therefore, we used available regulatory values for environmental quality standard. P*_i_*ave is the mean CF, and P*_i_*max is the maximum value of all metals in a particular site. PLI values were calculated from the CFs to minimize the possible anthropogenic influences.

Formula 1: $C_{f}^{i}= \frac{C^{i}}{C_{n}^{i}}$

Formula 2: $N\mathrm{PI} =\sqrt{\frac{P_{i avg}^{2}+ P_{i max}^{2}}{2}}$

Formula 3: PLI = ${(CF1 \times CF2 \times CF3 \times\ldots CFn)}^{1/n}$

According to Hakanson (1980), there are four levels of pollution classified: Cf < 1 (low contamination); 1 ≤ Cf < 3 (moderate contamination); 3 ≤ Cf < 6 (considerable contamination); Cf ≥ 6 (very high contamination). Iordache et al. (2022) and Su et al. (2022) classified Nemerow pollution index as: PI ≤ 0.7 (grade security), 0.7 < PI ≤ 0.1 (minimal impact), 1 < PI ≤ 0.1 (small impact), 2 < PI ≤ 0.1 (moderate impact), PI > 3 (heavy Impact), and PLI < 1 (unpolluted system), PLI > 1 (polluted system).

**References:**

Lau, E.V., Gan, S., & Ng, H.K. Extraction techniques for polycyclic aromatic hydrocarbons in soils. *Int. J. Anal Chem*. 398381 (2010).

Iordache, A.M., Nechita, C., Zgavarogea1, R., Voica, C., Varlam, M. & Ionete, R.E. Accumulation and ecotoxicological risk assessment of heavy metals in surface sediments of the Olt River, Romania. *Scientific Reports* **12**, 880 (2022).

Hakanson, L. An Ecological Risk Index for Aquatic Pollution Control a Sedimentological Approach. *Water Research* **14**, 975-1001 (1980).

Su, K., Wang, Q., Li, L. Cao, R. & Xi, Y. Water quality assessment of Lugu Lake based on Nemerow pollution index method. *Scientific Reports* **12**, 13613 (2022).
